# 560. Circulating *Pneumocystis jirovecii* Genotypes in Central-Eastern Canada

**DOI:** 10.1093/ofid/ofad500.629

**Published:** 2023-11-27

**Authors:** Valerie Roy, Anne Desjardins, Simon F Dufresne, Xavier Marchand-Senécal, Alex Carignan, Philippe J Dufresne

**Affiliations:** Université de Sherbrooke, Sherbrooke, Quebec, Canada; CHU de Québec, Quebec City, Quebec, Canada; CIUSSS de l'Est-de-l'Île-de-Montréal, Hôpital Maisonneuve-Rosemont, Montreal, Quebec, Canada; Hôpital Maisonneuve-Rosemont, CIUSSS de l'Est-de-l'Île-de-Montréal, Montréal, Quebec, Canada; Universite de Sherbrooke, Sherbrooke, QC, Canada; Laboratoire de Santé Publique du Québec, INSPQ, Sainte-Anne-de-Bellevue, Quebec, Canada

## Abstract

**Background:**

Genotyping allows description of *Pneumocystis jirovecii* (*Pjp*) heterogenous population structure and clusters linked to nosocomial transmission. It may also reveal mutations associated with resistance to antimicrobials. Recently, a multilocus sequence typing (MLST) scheme by *Pasic et al.* (2020) has been adopted by *International Society for Human & Animal Mycology* (ISHAM) as the typing method of choice for this fungus.

**Methods:**

We analyzed *Pjp* specimens submitted to the *Laboratoire de santé publique du Québec* for genotyping between February 2013 and March 2023. The loci genotyped were those used in the *Pasic et al.* scheme (mt26s, CYB, SOD, B-TUB) with the addition of dihydropteroate synthase (DHPS).

**Results:**

296 specimens from Central-Eastern Canada were genotyped: 266 from Quebec, 29 from Ontario, 1 from New Brunswick. Most specimens were submitted as part of outbreak investigations. 187 complete (161 unique and 26 mixed) and 109 incomplete genotypes were obtained. Among specimens with complete and unique genotypes, 87 isolates matched with 23 previously known sequence-types (ST), according to ISHAM MLST scheme. Twenty-four new ST with currently known alleles were described. We also identified 36 isolates with previously undescribed alleles which resulted in 21 new distinct ST. MLST typing allowed epidemiological description of 9 outbreaks. Out of 243 specimens for which DHPS amplification was possible, only one showed a mutation (DHPS 57) previously linked to trimethoprim-sulfamethoxazole resistance. This contrasts with high prevalence of mutant strains seen in some countries. ST-52 was the most prevalent with 32 isolates (20% of strains with complete and unique genotypes) and was involved in 3 outbreaks. A specific CYB mutation (G369T) conferring resistance to atovaquone was uncovered in 18 ST-52 isolates, all associated with an outbreak in one hospital.

**Conclusion:**

This is the first multicenter study to describe circulating genotypes of *Pjp* in Central-Eastern Canada. It represents the largest genotyping assembly performed according to the recent ISHAM MLST scheme. We described 45 new ST to be added to ISHAM’s 52 current genotypes. Our study highlights the usefulness of MLST typing in *Pjp* outbreak investigation and identification of potentially resistant isolates.

**Disclosures:**

**Simon F. Dufresne, MD**, AvirPharma Inc.: Grant/Research Support|Merck Canada Inc.: Grant/Research Support **Alex Carignan, MD, MSc**, GSK: Advisor/Consultant|GSK: Grant/Research Support|GSK: Honoraria|Moderna: Advisor/Consultant|Moderna: Honoraria|Orimed Pharma: Advisor/Consultant|Pfizer: Advisor/Consultant|Pfizer: Grant/Research Support|Pfizer: Honoraria

